# Information Seeking Source among Patient with Gastrointestinal Diseases and Functional Gastrointestinal Disorders

**Published:** 2018-06

**Authors:** Mohammad Hossein BIGLU, Morteza GHOJAZADEH, Samira KASHEFI

**Affiliations:** 1. Dept. of Basic Sciences, Faculty of Paramedical, Tabriz University of Medical Sciences, Tabriz, Iran; 2. Dept. of Physiology, Liver and Gastrointestinal Disease Research Center, Tabriz University of Medical Sciences, Tabriz, Iran; 3. Dept. of Medical Library and Information Sciences, School of Health Management and Medical Informatics, Tabriz University of Medical Sciences, Tabriz, Iran

## Dear Editor-in-Chief

Information seeking and acquisition play an important role in enabling a person to manage and cope with disease (1). Printed materials were least likely to be consulted first (2). Over the past decade, the use of the internet has increased. The internet was the most frequently used source (3). Study about seeking online health information of people with gastrointestinal diseases, show usage rates of between 42% and 92.6% (4–8). Various information needs and perceptions about usefulness are the reasons that patients prefer to seek information from various sources. The determining factors in selecting information resources are variability in specific needs, access to preferred sources (9), and easy access to sources (10). This paper focuses on participants’ perception usefulness source of health information.

Questionnaires were distributed at Sheykhoreis Clinic, Emam Reza Hospital, Tabriz, Iran and other gastroenterology clinics in Tabriz City over a three-week period in July 2016. From 180 participants, 45.6% were male. Age range of participants was between 17 and 47 yr, averaging 32.84. About patients educational level, 16.8% had some high school and at a lower level, 33.7% had a high school degree, and 49.4% had a college degree. Among the participants, 71.7% had a computer at home and 85% (n=153) accessed the internet from home or via a mobile network. The average time of using the internet was 2:30 hours.

In order to determine the participants’ perceived usefulness of the information source, they were asked to rate how useful different types of sources had been in helping them to find out what they needed to know about their disease (Table 1). The participants rated physicians and dieticians as the most useful source. Among the media, the internet was rated as the most useful source. Within mass media, television was preferred. Search engines and medical websites were rated the most useful among internet sites. Cooking and recipes were rated as the most useful topics. The patients also rated disease management, disease prevention, disease-related complications, and diet as the most important subjects for controling their condition.

**Table 1:** Usefulness of different types of sources among 180 patients

***Sources***		***N***	***M***
Person	Physicians	161	4.05
	Dieticians	54	3.56
	Family members with GI & FGID	81	3.44
	Other people with GI & FGID	65	3.26
	Friends with GI & FGID	62	3.26
	Pharmacists	62	3.24
	Family members don’t have GI & FGID	76	3.21
	Nurses	51	3.02
	Friends don’t have GI & FGID	63	2.95
	Counselors/social workers	36	2.78
	Librarians	33	2.33
Media	Internet	138	4.21
	Television	103	3.76
	Journals	62	3.53
	Books	76	3.50
	Brochures/Pamphlets	56	3.30
	Magazines	51	2.82
	Radio	52	2.71
	Newspapers	49	2,61
Internet Site	Search engines	124	4.11
	Medical Websites	62	4.05
	General news Websites	64	3.95
	Personal Websites	50	3.34
	Government agency Websites	41	3.32
	Blogs	60	2.93
Topics	Cooking/recipes	93	4.32
	Disease management	116	4.14
	Disease prevention	119	4.11
	Disease-related complications	86	4.10
	Diet	83	4.04
	Diagnostic tests	78	4.03
	Exercise	76	3.97
	Treatment options	90	3.78
	Medication options, side effects, and interactions	79	3.76
	Signs/symptoms	111	3.75
	Risk factors	65	3.69
	Vitamins/supplements	63	3.62
	Medication warnings /allergies	70	3.40

Patients with gastrointestinal diseases and functional gastrointestinal disorders want to control their condition as well as to improve their health and quality of life, therefore, awareness about health issues has increased and information sources have an important role in the prevention and diagnosis of diseases. Physicians, nurses, and dieticians should allocate enough time to educate patients about their condition and related information needed. The participants use the internet more than other media.

The internet is suitable for physician and patient communication in order to renew prescriptions and answer questions. Therefore, it is worthwhile for hospitals and clinics to design websites that present useful medical information for patients to resolve their lack of information.
